# Draft genome sequence of *Bacillus thuringiensis* 147, a Brazilian strain with high insecticidal activity

**DOI:** 10.1590/0074-02760150273

**Published:** 2015-09

**Authors:** Luiz Carlos Bertucci Barbosa, Débora Lopes Farias, Isabella de Moraes Guimarães Silva, Fernando Lucas Melo, Bergmann Morais Ribeiro, Raimundo Wagner de Souza Aguiar

**Affiliations:** 1 Universidade Federal de Itajubá, Instituto de Recursos Naturais, Itajubá, MG, Brasil; 2 Universidade Federal do Tocantins, Departamento de Engenharia de Bioprocessos e Biotecnologia, Gurupi, TO, Brasil; 3 Universidade de Brasília, Departamento de Biologia Celular, Brasília, DF, Brasil

**Keywords:** Bacillus thuringiensis, biopesticides, genome sequence

## Abstract

*Bacillus thuringiensis *is a ubiquitous Gram-positive and sporulating bacterium. Its crystals and secreted toxins are useful tools against larvae of diverse insect orders and, as a consequence, an alternative to recalcitrant chemical insecticides. We report here the draft genome sequence of*B. thuringiensis *147, a strain isolated from Brazil and with high insecticidal activity. The assembled genome contained 6,167,994 bp and was distributed in seven replicons (a chromosome and 6 plasmids). We identified 12 coding regions, located in two plasmids, which encode insecticidal proteins.

*Bacillus thuringiensis* is a Gram-positive bacterium that has been isolated from a range of ecosystems including soil, water and dead insects, among others. *B. thuringiensis* is a spore-forming bacterium that synthesises parasporal crystalline inclusions containing Cry and Cyt proteins (also known as δ-endotoxins) and some of these are toxic against a wide range of insect orders, nematodes and human cancer cells (Palma et al. 2014). *B. thuringiensis* isolates can also synthesise and secrete other insecticidal proteins during the vegetative growth phase, which are designated vegetative insecticidal proteins (Vip) and secreted insecticidal protein (Sip). Furthermore, other predicted toxins are also produced by *B. thuringiensis* strains, but their toxicity has yet to be proven (Palma et al. 2014).

The crystals and secreted toxins of *B. thuringiensis* are highly specific for their hosts and have therefore gained worldwide importance as an alternative to chemical insecticides, motivating the search for new *B. thuringiensis*isolates to identify and characterise new insecticidal proteins (Pardo-López et al. 2013, Palma et al. 2014). Accordingly, whole genome sequence of these isolates can be an important starting point. In this study, we determined the draft genome sequence of*B. thurin-giensis* 147, a strain isolated from soil samples in the state of Tocantins, Brazil. Toxicity assays of this strain have shown high insecticidal activity against larvae from* Aedes aegypti* (Diptera: Culicidae) and*Spodoptera frugiperda* (Lepidoptera: Noctuidae).

For genome sequencing, total DNA (chromosome and plasmids) was isolated using the Wizard Genomic DNA Purification kit (Promega) from fresh overnight cultures. Whole-genome sequencing was performed with the MiSeq platform (Illumina, USA), located at the High-Performance Genome Centre of Federal District (Brasília, Brazil) using the 600-cycle MiSeq reagent kit v.3 (Illumina). A total of 2,614,978 paired-end reads were generated at a read length of 150 bp. A quality control of these reads was performed with the FastQC tool (bioinformatics.babraham.ac.uk/projects/fastqc/). *De novo* genome assembly was carried out with SPAdes 3.5.0 (Bankevich et al. 2012). The final draft genome assembly consisted of 94 contigs (length > = 500 bp), with a total size of 6,167,994 bp, N50 value of 205,568 and a mean guanine-cytosine content of 34.90%. A BLAST analysis (blast.ncbi.nlm.nih.gov/blast/Blast.cgi) of each contig showed that the assembled genome was distributed in seven replicons: a circular chromosome and six plasmids. The genetic information about these replicons is summarised in Table I.

**TABLE I:** Genetic information about the replicons from *Bacillus thuringiensis* 147

Replicon	Length (bp)	GC content (%)	Predicted genes (n)
Chromosome	5,337,997	35.07	5,602
Plasmid 1	357,957	32.32	355
Plasmid 2	5,053	34.93	3
Plasmid 3	235,436	36.59	267
Plasmid 4	217,152	32.99	219
Plasmid 5	7,640	35.33	8
Plasmid 6	6,759	35.61	3

GC: guanine-cytosine.

Automated annotation, carried out using the RAST annotation server (Aziz et al. 2008), showed that the draft genome of *B. thuringiensis* strain 147 contains 6,319 predicted protein-coding sequences and 138 predicted RNAs (rRNAs and tRNAs). These data are consistent with other published complete genomes from *B. thuringiensis* strains (Doggett et al. 2013, Liu et al. 2014, Johnson et al. 2015). In addition to the analysis performed by the RAST annotation server, the identification and annotation of insecticidal genes were performed with BLAST (Altschul et al. 1997), using a custom insecticidal toxin database from *B. thuringiensis*. The local custom database was constructed with amino acid sequences of δ-endotoxins (Cry and Cyt), secreted toxins (Vip and Sip), proteins called “mosquitocidal toxin” and haemagglutinin-related proteins, all retrieved from the curated UniProtKB database (uniprot.org/uniprot/). All insecticidal proteins identified using a local database were confirmed using remote BLAST (blast.ncbi.nlm.nih.gov/blast/Blast.cgi). Insecticidal genes were confined to two plasmids. Plasmid 1 and plasmid 4 were found to harbour one and 11 insecticidal genes, respectively. Table II summarises the regions of the assembled contigs that encode insecticidal proteins.

**TABLE II:** Predicted regions that encode insecticidal proteins (or fragments of these)

Replicon	Contig	Start	End	Strand	BLAST description
Plasmid 1	88	21,036	22,583	+	Mosquitocidal toxin protein
Plasmid 4	10	5,906	5,115	-	Type-2Ba cytolytic delta-endotoxin
	20	12,527	11,616	-	Cry protein
	45	2,814	4,841	+	Pesticidal crystal protein Cry10Aa
	53	6,603	6,313	-	Pesticidal crystal protein Cry4Aa
	8	5	261	+	Pesticidal crystal protein Cry4Ba
	84	115	295	+	Pesticidal crystal protein Cry4Ba
	84	2,719	1,142	-	Toxin protein/Cyt-like protein
	9	13,868	13,119	-	Type-1Aa cytolytic delta-endotoxin
	9	15,535	17,466	+	Pesticidal crystal protein Cry11Bb
	9	18,651	18,776	+	Pesticidal crystal protein Cry28Aa
	92	1,551	136	-	Haemagglutinin-related protein

This Whole Genome Shotgun project has been deposited at DDBJ/EMBL/GenBank under the accession LFXM00000000. The version described in this paper is version LFXM01000000.
